
JOURNAL INFORMATION
==============================
Journal ID: Intereconomics

Intereconomics

EISSN: 1613-964X

ARTICLE INFORMATION
==============================
PMCID: PMC8992411
DOI: 10.1007/s10272-022-1030-0
Article ID: 1030
Article version: 1
Subjects: Editorial

The EU Without Russian Oil and Gas

Wolff Guntram B. guntram.wolff@bruegel.org

grid.432713.0 Bruegel, Rue de la Charité 33, 1210 Brussels, Belgium
Electronic publication date: 2022 Apr 8
Print publication date: 2022
Volume: 57
Issue: 2
First page: 66
Last page: 67
Copyright: © The Author(s) 2022
License: This article is distributed under the terms of the Creative Commons Attribution 4.0 International License (https://creativecommons.org/licenses/by/4.0/).
Open Access funding provided by ZBW — Leibniz Information Centre for Economics.
License URL: https://creativecommons.org/licenses/by/4.0/

Keywords: F51
issue-copyright-statement: © The Author(s) 2022

==============================

DOI: 10.1007/s10272-022-1030-0

*Intereconomics*, 2022, 57(2), 66-67

JEL: F51

### The EU Without Russian Oil and Gas

Following the Russian invasion and brutal attack on Ukraine, the West and in particular the United States, the United Kingdom, Canada and the EU have agreed on a number of major and far-reaching sanctions. And while these sanctions had strong effects on the Russian economy, they are falling short of their goal of ending Russia's attack and bringing its economic capacity for war to an end. Further sanctions on Russian oil and gas need to be discussed.

The financial sanctions against Russia since its invasion of Ukraine began on 24 February have included three main planks, as Nicolas Véron and Joshua Kirschenbaum point out.[1] First, sanctions against named Russian individuals have expanded dramatically. Second, a series of sanctions have been imposed on individual Russian banks. These include the disconnection of individual Russian banks from SWIFT, the international interbank messaging system which is based in Belgium and thus under EU jurisdiction. Third, the Bank of Russia, the country's central bank, has been blocked from using its international reserves in several jurisdictions. Critically, these include the US, the EU, the UK, Canada, Japan, Australia and Switzerland – in other words, all the world's core reserve-currency jurisdictions bar China.

A second set of sanctions concerns technological products. Targeted sanctions on specific technologies, financial sanctions and "self-sanctioning" by private companies are effectively decoupling Russia from supplies of high-tech goods. The combination of technological and financial sanctions, public pressure and reputational risk, and the collapse of the Russian economy has made the decision to leave the Russian market easy for companies, and not just those from NATO allied countries.

Russia is highly reliant on imports of high-tech goods, with imports worth around $19 billion annually. The largest share (45%) comes from the EU, with 21% from the US, 11% from China and 2% from the UK. Most nuclear technology imports in 2019 came from the EU (68%). The EU is also the main provider of biotechnology, electronics, life sciences and flexible manufacturing goods. Russia has tried to resist technological sanctions by import substitution, but without much success. High-tech products are developed using inputs from many countries, but few of them can function without inputs from the EU or the US. In some high-tech goods industries, the effects of sanctions are already being felt. In the long term, sanctions will also severely affect Russia's growth outlook and ensure that waging war means Russia will cease to be a modern economy.[2]

These sanctions have had a strong effect on the Russian rouble. In fact, the currency initially dropped massively by almost 50% and even Russia acknowledged in early March that the sanctions were constituting a significant blow to its economy.[3] However, the exchange rate recovered substantially, undoing more than half of the initial loss.

An important reason why, despite all these sanctions, the rouble could recover and the Russian economy did not completely implode is the continued stream of revenues from oil, gas and coal sales. In fact, Russia remains the world's first exporter of oil and gas, and at current energy prices this provides large hard currency revenues, estimated at around $700 million per day for crude oil and refined products and $400 million per day for piped natural gas to the EU alone.

1 https://www.bruegel.org/2022/03/war-in-europe-the-financial-front/.
2 https://www.bruegel.org/2022/03/the-decoupling-of-russia-high-tech-goods-and-components/.
3 https://edition.cnn.com/2022/03/02/business/russia-markets-economy-sberbank/index.html.

© The Author(s) 2022. Open Access: This article is distributed under the terms of the Creative Commons Attribution 4.0 International License (https://creativecommons.org/licenses/by/4.0/).

Open Access funding provided by ZBW – Leibniz Information Centre for Economics.

While the US, Canada and the UK have announced embargoes or phase-out measures for Russian energy in the wake of the war in Ukraine, the EU has held back, instead launching a new energy strategy, REPowerEU. This aims to reduce the EU's gas imports from Russia by nearly two-thirds by the end of 2022, and to make Europe independent from all Russian fossil fuels well before 2030. However, such a partial and gradual wind-down of volumes from Russia is ineffective. There is a risk that this strategy will drive up prices even further, over-compensating Russia for the loss of volume.

The current sanction strategy is therefore not effective enough to meaningfully change the calculations of Russian leadership. And as Russian economists Sergei Guriev and Oleg Itskhoki point out, the continued revenues from oil and gas sales are used by President Putin to finance his brutal war in Ukraine.[4] In fact, the revenues from fossil fuel sales are so high that they can likely solve Russia's fiscal and balance of payment problems. The authors point out that the pre-war budget was balanced at an oil price of $44 per barrel. Without the external revenues coming from the sale of fossil fuels, Russia would likely run a substantial fiscal deficit. It is true that Russia can print roubles to close the deficit. However, already now, the inflation rate has massively increased and the loss of hard currency revenues would likely result in a further increase in inflation. Put differently, the salaries of Putin's police and military would also lose value if fossil fuel revenues dried up.

Numerous voices therefore call on EU leaders to follow the US, UK and Canada and implement a full embargo on imports of Russian fossil fuels and gas. However, German Chancellor Olaf Scholz rejected the embargo and stated that this represents a conscious decision by European governments, as these imports are of essential importance for the everyday life of European citizens.

An immediate EU fossil fuel embargo would undoubtedly imply substantial costs. However, a group of economists have argued that these costs are still manageable in size, quite comparable to the fallout from the COVID-19 pandemic.[5] This is not the place to discuss the paper and the subsequent controversy around it. It should be noted, however, that an important reason why the cost is limited relates to adjustments, or substitution effects. The German Chancellor's reasoning, that entire economic sectors would be affected or even stopped, is therefore an inaccurate description of the overall effect of a full embargo as the industrial structure of Germany would shift, adjusting to the energy price shock. In fact, I would argue that it would make sense to accept such a sectoral change in the composition of Germany's economy. Reducing its reliance on cheap fossil energy may be necessary and specialising in relatively energy-intensive industries with large export potential may be an economic model that has run its course.

Nevertheless, a full embargo would be a huge and far-reaching step. And while it would hit Putin hard in the short term, it would also accelerate the adjustment process in Russia towards different sources of demand for its fossil fuels. Currently, the physical infrastructure is not there to bring the oil and gas to energy-hungry Asia. Still, even with the infrastructure in place, Putin would likely lose – even in the long term – as China will exploit its unique position as the main remaining buyer of Russian fossil fuel to reduce the price.

Overall, a more sensible approach may therefore be for the EU to impose price caps or even tax energy imports from Russia. An import tariff as recently proposed by Ricardo Hausmann could go a long way towards reducing the major source of revenues to the Russian economy and to Putin himself.[6] The EU and the West need to acknowledge and accept the fact that European liberal democracy is being defended in Ukraine – a clearer message to Putin is needed. A slow and gradual phasing out of Russian fossil fuel is simply neither ethically acceptable nor politically and economically smart.

**Guntram Wolff**, Bruegel, Brussels, Belgium.

4 https://www.dropbox.com/s/wmptre3vkfkqysf/Guriev%20Itskhoki.pdf?dl=0.
5 https://www.econtribute.de/RePEc/ajk/ajkpbs/ECONtribute_PB_028_2022.pdf.
6 https://www.project-syndicate.org/commentary/case-for-punitive-tax-on-russian-oil-by-ricardo-hausmann-2022-02.
